# Dentistry a Worthy Profession

**Published:** 1906-08-15

**Authors:** S. H. Guilford


### DENTISTRY A WORTHY PROFESSION.

BY PROF. S. H. GUILFORD.

(The following is an extract from the Commencement Address to the graduates of the Philadelphia Dental College

Dentistry is essentially a branch of the healing art, probably coeval with medicine and of similar import. It came into existence in response to the cry for relief from pain. Its origin was lowly, but not more so than medicine.

All of the arts and sciences had their foundations laid in a crude and imperfect manner. Architecture had its beginning in the bending and binding together of the saplings of the forest to afford a shelter for primeval man. Designing probably had its inception in the tracing of forms or figures in the sand on the shore of lake or sea. Painting in its infancy must have consisted in the use of colored earths laid upon stone or cliff side to reproduce the mind picture of the aborigine, and sculpture may have begun with the rude shaping of clay into the forms of animated beings.

A lowly beginning has been the lot of many vocations that have in time grown into useful and æsthetic aids to the development or refinement of mankind. It is the subsequent progress that any craft or occupation may make and its influence upon the world's betterment that must be the measure of its value.

Judged by this standard has dentistry advanced in keeping with other arts and sciences?

Dentistry may be considered as twin brother to surgery and the relation was early recognized in the naming of our specialty "dental surgery."

Each in its effort to relieve suffering or possible death was obliged to remove a part or the whole of an organ and the mutilation thus caused naturally turned attention to a substitution of the lost part by artificial means.

Such artificial substitution in both surgery and dentistry has been gradually perfected through the ingenuity of man until the part supplied has been made to serve the purpose of the lost member in a remarkably efficient manner.

In the course of time man grew sensitive to his mained condition and naturally desired to conceal it. A higher type of art then came into play and attention was directed to the concealment of artificiality. Surgery has made notable progress along this line, but dentistry has far outstripped it probably because the need of it seemed greater.

At this point the two branches of surgery seemed to come to the parting of the ways, for while surgery proper has been greatly aided in its beneficient designs by the assistance of anæsthesia, antisepsis and scientific nursing, in all of which dentistry has shared the benefit, our vocation has developed principally along other lines.

While it is not within the province of surgery to prevent the accidents or diseases which call for the exercise of its skill, it was found to be distinctly within the field of dentistry to arrest by skillful mechanical means the ravages of those diseases which attacked the organs of the oral cavity.

During more than a century this checking the course of caries has attained to greater and still greater perfection until the results achieved are not only wonderful in themselves, but of the greatest possible benefit to mankind. Not satisfied, however, with even such accomplishments, dentistry felt impelled to seek out the cause of tooth impairment in order that, in a measure at least, it might be prevented. If reparation of lost tissue is a praiseworthy achievement, certainly its prevention is a greater and more beneficent one.

We hear much in these days of preventive medicine and its results can hardly be overestimated or too highly praised, but let us not forget that preventive dentistry has also made wonderful strides and is rapidly advancing day by day.

To day, the chief glory of dentistry lies, not in the sacrifice of important organs but in their conservation, thus adding both to the health and comfort of the human race. Experiment, investigation and tentative practice are exhibiting results in the way of prevention scarcely dreamed of even by our immediate predecessors.

This has been made possible by the persistent education of the public to the importance of preserving the teeth and especially in impressing upon the mothers who come under our care the absolute necessity of dental oversight of their children. So well has this been done that the little ones are brought to us for advice and attention long before their dental organs have been attacked by any disorders.

The good results of this practice are already seen, but they will be more apparent in the coming generation, for it is hardly prophetic to say that within present knowledge and faithful oversight dental affections of every kind must diminish. With the dental organs preserved in a healthy condititions they will not only be retained and be serviceable even to old age, but the factor of pain from which even the greatest of the world's benefactors have suffered will be eliminated.

Such faith have I in the efficacy of preventive measures that I do not hesitate to declare that the child is already born who barring accident or systemic disease, will never know the meaning of dental discomfort.

While dentistry is justly proud of what it has achieved in the relief of suffering, in artificial substitution, in checking the ravages of decay and later in its partial prevention, the natural development of its possibilities has led it into another field where the æsthetic holds sway.

If the human face in its ideal form reflects the image of its Maker and the play of the features is capable of outward expression to the loftiest thoughts and emotions of mind and soul, how greatly is this power modified or when deformity or malformation exists? Besides, what more beautiful than the human face when its component parts are in proper proportion and harmonious relation, and what more unattractive or unpleasing when harmony is lacking?

The field of dental operation is limited to but part of the face, but it is the portion that is most subject to malformation or derrangement, and with half disarrangement even approximate harmony is impossible.

The comparatively new branch of dental practice, known as orthodontia, has been developed for the express purpose of preventing and correcting not only any malarrangement of the teeth to promote their greater usefulness, but also to obliterate any deformities of the teeth and jaws and thus restore the harmonious relations of the features.

Surely this is the highest surgical art of which we have any conception at present. To take an unattractive face and by mechanical means, and almost without pain mould it into an attractive and even beautiful form is an achievement worthy of the greatest praise.

Yes, dentistry has invaded the field of art to some purpose and who shall say to what heights it may yet attain. Have we not reason to feel proud of the benefit it has conferred upon mankind and is it not a pleasure to feel that our skill and earnest efforts have been appreciated by a grateful public?—*Stomatologist.*

